# Learning and Growing Together: More Fully Integrating Research, Practice, and Organizational Policy

**DOI:** 10.1093/geroni/igaf122.3479

**Published:** 2025-12-31

**Authors:** Lea Efird-Green, Jennifer Terray, Heidi Gil, Sheryl Zimmerman

**Affiliations:** The University of North Carolina at Chapel Hill, Chapel Hill, North Carolina, United States; LiveWell, Plantsville, Connecticut, United States; LiveWell, Plantsville, Connecticut, United States; University of North Carolina at Chapel Hill, Chapel Hill, North Carolina, United States

## Abstract

Community-engaged research is important if results are to be relevant to end-users and inform practice and policy; however, the extent to which such research truly partners with community members is highly variable. This project expanded a partnership that developed two “living well with dementia” measures, such that the community partner was fully engaged in actually designing and conducting the evaluation of those measures; in this way, the extent to which the partner functioned as the researcher and informed the research process constitutes a new model for community-engaged research. Process findings indicated full partnership requires that (1) the mission of the partner be fully aligned with and supported by the focus of the research (in this case, LiveWell, a dementia-focused services organization committed to a strengths-based approach to supporting the wellbeing of people living with cognitive change or dementia); (2) the partner be invested in learning about and contributing to the research process (including the development of pragmatic study protocols); (3) the researcher be open to developing protocols that extend beyond traditional research (e.g., being willing to solicit IRB-approval that confidential information may be incorporated into the partner’s care plans – notably, to date nearly 80% of consents have included permission for this sharing); and (4) the researcher allow that the protocol may expand to include participants ineligible for study. This presentation will detail these and other novel findings to serve as a model of research that benefits community partners and researchers and has true promise to inform practice and policy.

